# Isorhamnetin attenuates osteoarthritis by inhibiting osteoclastogenesis and protecting chondrocytes through modulating reactive oxygen species homeostasis

**DOI:** 10.1111/jcmm.14333

**Published:** 2019-04-14

**Authors:** Feng Zhou, Jingtian Mei, Kai Yuan, Xiuguo Han, Han Qiao, Tingting Tang

**Affiliations:** ^1^ Shanghai Key Laboratory of Orthopaedic Implants, Department of Orthopaedic Surgery Shanghai Ninth People's Hospital, Shanghai Jiao Tong University School of Medicine Shanghai P. R. of China

**Keywords:** apoptosis, isorhamnetin, osteoarthritis, osteoclast, ROS

## Abstract

Increasing evidence indicates that osteoarthritis (OA) is a musculoskeletal disease affecting the whole joint, including both cartilage and subchondral bone. Reactive oxygen species (ROS) have been demonstrated to be one of the important destructive factors during early‐stage OA development. The objective of this study was to investigate isorhamnetin (Iso) treatment on osteoclast formation and chondrocyte protection to attenuate OA by modulating ROS. Receptor activator of nuclear factor‐kappa B ligand (RANKL) was used to establish the osteoclast differentiation model in bone marrow macrophages (BMMs) in vivo. H_2_O_2_ was used to induce ROS, which could further cause chondrocyte apoptosis. We demonstrated that Iso suppressed RANKL‐induced ROS generation, which could mediate osteoclastogenesis. Moreover, we found that Iso inhibited osteoclast formation and function by suppressing the expression of osteoclastogenesis‐related genes and proteins. We proved that Iso inhibited RANKL‐induced activation of mitogen‐activated protein kinase activation of mitogen‐activated protein kinase (MAPK), nuclear factor‐kappa B (NF‐κB) and AKT signalling pathways in BMMs. In addition, Iso inhibited ROS‐induced chondrocyte apoptosis by regulating apoptosis‐related proteins. Moreover, Iso was administered to an anterior cruciate ligament transection (ACLT)‐induced OA mouse model. The results indicated that Iso exerted beneficial effects on inhibiting excessive osteoclast activity and chondrocyte apoptosis, which further remedied cartilage damage. Overall, our data showed that Iso is an effective candidate for treating OA.

## 1 INTRODUCTION

As the most prevalent form of arthritis, osteoarthritis (OA) affects millions of people, leading to disability.^1^, ^2^ Knee joints, especially in the elderly, are the most common site of OA.^3^ In addition to pain, OA may cause joint stiffness and limited joint motion.^4^ Conservative treatments such as non‐steroidal anti‐inflammatory drugs (NSAIDs) merely relieve OA progression temporarily.^5^ Joint replacement is considered a definitive treatment for patients with advanced/end‐stage OA.^6^ Considering surgical risks and economic costs, it is necessary to further investigate this disease to find new potential treatment strategies.

OA is characterized by progressive loss of articular cartilage, remodelling of the subchondral bone and osteophyte formation.^7^, ^8^ Currently, increasing evidence has shown that osteoclast activity and bone remodelling increase in the early stage of OA, disturbing the equilibrium between bone formation and resorption that could eventually lead to a marked reduction in subchondral bone thickness.^9^, ^10^ Moreover, the imbalance between bone formation and resorption leads to abnormal reconstruction of subchondral bone, which could promote the progression of OA.^11^ This evidence provides a theoretical basis for targeting osteoclasts in the early stage of OA, which could be a useful intervention strategy for the treatment of OA.

Increasing evidence shows that reactive oxygen species (ROS) are one of the important destructive factors during OA development.^12^ ROS perform various functions in cell energetic cycling, extracellular matrix metabolism and maintenance of chondrocyte homeostasis under physiological conditions. However, redundant production of ROS will break normal signalling and homeostasis.^13^ Increased production of ROS is frequently found in OA, not only promoting osteoclastogenesis but also leading to matrix metalloproteases (MMPs) secretion and degradation of cartilage.^14^, ^15^, ^16^ Moreover, ROS can induce dysregulation of mitochondria by depolarizing the mitochondrial membrane, which further results in increased ROS production^17^, ^18^ and causes a vicious circle. Numerous studies have shown that oxidative stress caused by ROS can induce apoptosis in chondrocytes.^19^, ^20^ All of these findings highlight that ROS modulation may be a potential treatment for OA by inhibiting osteoclastogenesis and protecting chondrocytes.

Isorhamnetin (Iso), a 3′‐methylated flavonol derived from herbal medicinal plants, has been shown to have a significant impact on many pathological processes. Iso was reported to protect against hypoxia/reoxygenation‐induced injury by attenuating oxidative stress in cardiomyocytes.^21^ Iso was also capable of inhibiting cancer cells migration, indicating that it could be a potential clinical chemotherapeutic approach for cancer.^22^ Furthermore, Iso has been shown to be an effective compound that reduced IL‑1β‑induced expression of inflammatory mediators in human chondrocytes.^23^ Despite these early reports indicating the translation potential in pre‐clinical research, whether Iso could be used to treat OA by inhibiting osteoclasts and reducing ROS‐induced chondrocyte apoptosis remains largely unexplored.

In the present study, we used receptor activator of nuclear factor‐kappa B ligand (RANKL) to activate osteoclasts to mimic the pathological environment in the early stage of OA. We investigated the effects of Iso treatment on RANKL‐induced osteoclast activation. Additionally, the underlying mechanisms regarding Iso‐induced inhibition of osteoclastogenesis were investigated. Moreover, H_2_O_2_ was used to induce ROS generation in chondrocytes and we investigated whether Iso could attenuate ROS‐induced chondrocyte apoptosis. For in vivo studies, Iso was administered to an anterior cruciate ligament transection (ACLT)‐induced OA mouse model. We aimed to provide novel insights regarding OA treatment and a foundation for future translational use of Iso in the clinic.

## 2 MATERIALS AND METHODS

### 2.1 Cells, media and reagents

Primary bone marrow macrophages (BMMs) were cultured in α‐minimum essential medium (α‐MEM, HyClone, Logan, UT) containing 10% foetal bovine serum (Gibco, New York), 100 U/mL penicillin and 100 μg/mL streptomycin (Gibco, New York) at 37°C in a humidified atmosphere with 5% CO_2_. Primary chondrocytes were cultured with Dulbecco's minimum essential medium (DMEM; HyClone, Logan, UT) containing 10% foetal bovine serum, 100 U/mL penicillin and 100 μg/mL streptomycin under the same conditions as BMMs. Iso was purchased from Herbpurify (Chengdu, China). RANKL and M‐CSF were purchased from PeproTech (Rocky Hill, NJ). Antibodies against nuclear factor‐kappa B (NF‐κB), AKT and mitogen‐activated protein kinase (MAPK) were purchased from Abcam (Cambridge, MA). H_2_O_2_ solution was obtained from Beyotime Biotechnology (Shanghai, China) and kept at 4°C while being protected from light.

### 2.2 BMMs isolation and culture

BMMs were obtained from the long bones of 6‐week‐old C57/BL6 mice. Briefly, after killing, the long bones, including femurs and tibias, were separated under aseptic conditions. All of the bone marrow was flushed from mouse femurs and tibias and then resuspended in complete α‐MEM containing 30 ng/mL M‐CSF. Cells were cultured in 75‐cm^2^ flasks and cultured in a 5% CO_2_ incubator at 37°C. The media were changed every other day to remove non‐adherent cells. At 80% confluence, the cells were washed with PBS three times and then trypsinized to harvest BMMs for the following experiments.

### 2.3 Primary chondrocytes culture and immunofluorescence staining

Male C57BL/6J mice were killed and disinfected using 75% alcohol for 10 minutes. The head of the femur was exposed under aseptic conditions. All articular cartilage was isolated, collected and cut into 1 mm^3^ pieces. The tissue was digested with 0.25% trypsin and 0.2% collagenase II for 30 minutes and 5 hours respectively. The cells were then filtered through a 70‐μm cell strainer and washed three times with PBS. Next, the collected cells were seeded into culture dishes in DMEM at 37°C and 5% CO_2_. The culture medium was changed every other day. The expression of collagen II in chondrocytes after Iso treatment was analysed by immunofluorescence staining using collagen II antibody (Santa Cruz Biotechnology, CA).

### 2.4 Cell viability assay

To evaluate the cytotoxic effects of Iso, cell viability was analysed using a cell counting kit‐8 assay (CCK‐8, Dojindo, Japan) following the manufacturer's protocol. In the in vitro assay, BMMs that were cultured with α‐MEM containing 30 ng/mL M‐CSF and chondrocytes were treated with varying concentrations of Iso (0, 3.125, 6.25, 12.5, 25, 50, 100 and 150 μmol/L) for 24 hours. Next, 10% CCK‐8 solution was added to each well. The cells were cultured in a 37°C, 5% CO_2_ incubator for an additional 2 hours. Then, the absorbance was measured at a wavelength of 450 nm using a microplate reader (Bio‐Rad, Hercules, CA). Cell viability relative to the control group was calculated according to a previous study.^24^

### 2.5 In vitro osteoclastogenesis and bone resorption assay

To induce osteoclasts, BMMs harvested as mentioned above were seeded in a 96‐well plate at a density of 10^4^ cells/well. After 12 hours, the cells were treated with 50 ng/mL RANKL and 30 ng/mL M‐CSF for osteoclast differentiation according to a previous study.^25^ Moreover, varying concentrations of Iso were added and the media were changed every other day for 7 days when osteoclast formation was observed. The cells were fixed with 4% paraformaldehyde for 30 minutes and then diagnostic acid phosphatase staining was used to detect tartrate‐resistant acid phosphatase (TRAP) activity. Osteoclasts were identified as TRAP‐positive cells that contained more than three nuclei. The numbers of TRAP‐positive cells in each well were using Image‐Pro Plus software (Media Cybernetics, Bethesda, MD).

Bovine bone slices were placed in a 96‐well plate with BMMs 10^4^ cells/well seeded on top of the slices and the cells were cultured in α‐MEM containing 50 ng/mL RANKL and 30 ng/mL M‐CSF. The cells were further treated with different concentrations of Iso (0, 6.25, 12.5 or 25 μmol/L) until mature osteoclasts formed. With the assistance of mechanical agitation and sonication, the slides were washed with PBS to remove the cells. Resorption pits were imaged using scanning electron microscopy (SEM; FEI Instr., Hillsboro, OR).

### 2.6 Quantitative real‐time polymerase chain reaction (qRT‐PCR) analysis

BMMs were seeded in a six‐well plate at a density of 10^5^ cells/well and cultured with complete α‐MEM medium containing 50 ng/mL RANKL and 30 ng/mL M‐CSF. The cells were treated with different concentrations of Iso (0, 6.25, 12.5 or 25 μmol/L) until mature osteoclasts formed. Chondrocytes were treated with H_2_O_2_ and different concentrations of Iso (0, 6.25, 12.5 or 25 μmol/L) for 24 hours. Total RNA was extracted using TRIzol reagent (Invitrogen, CA). cDNA synthesis was performed with TaqMan reverse transcription reagent (Applied Biosystems, Foster, CA, USA) and transcription‐PCR was performed with real‐time PCR (ABI 7500; Applied Biosystems, Foster City, CA) according to a previous study.^26^ The primers that were used are listed in Table S1. The values were normalized to GAPDH mRNA expression levels and calculated using the 2^‐ΔΔCt^ method.

### 2.7 Western blotting

Western blotting analysis was performed according to a previously published protocol.^27^ Total protein was extracted from BMMs and chondrocytes using radioimmunoprecipitation assay buffer (RIPA; Millipore, MA) containing protease and phosphatase inhibitors. The protein concentrations were analysed using a BCA protein assay kit. A total of 20 μg of protein was separated using 10% SDS‐PAGE gel electrophoresis and then transferred to a polyvinylidene fluoride (PVDF) membrane. The membranes were blocked with 5% non‐fat milk for 2 hours and incubated overnight at 4°C with primary antibodies. Furthermore, the membranes were incubated with secondary antibodies for 1 hour and washed in Tris‐buffered saline with Tween (TBST). Fluorescent signals were detected using an Odyssey imaging system (Li‐Cor, Lincoln, NE).

### 2.8 Cell cycle analysis

Chondrocytes were treated with or without 25 μmol/L Iso for 24 hours. Cell cycle analysis was performed by propidium iodide (PI) staining followed by analysis with a FACScan flow cytometer (BD, CA).

### 2.9 Intracellular ROS detection

Levels of ROS in BMMs and chondrocytes were measured using DCFH‐DA probe according to a previous study.^28^ BMMs were cultured with or without 50 ng/mL RANKL and 30 ng/mL M‐CSF as well as different concentrations of Iso for 24 hours. Chondrocytes were treated with or without H_2_O_2_ and different concentrations of Iso for 24 hours. A final concentration of 10 µmol/L DCFH‐DA was added. After incubation in the dark for 20 minutes at 37°C, the cells were washed three times with PBS. Fluorescent signals were observed using fluorescence microscopy and the number of ROS‐positive cells in each field were then calculated.

### 2.10 Flow cytometry apoptosis analysis

For annexin V/propidium iodide (PI) apoptosis analysis, chondrocytes (5×10^5^/well) were seeded in 6‐well plates and cultured for 12 hours, followed by culturing with or without H_2_O_2_ (200 μmol/L) and various concentrations of Iso for 24 hours. After washing with PBS three times, chondrocytes were digested in 0.25% trypsin at 37°C and resuspended in binding buffer followed by annexin V‐FITC/PI staining (BD, CA) according to the manufacturer's instructions. The samples were analysed using a FACScan flow cytometer.

### 2.11 Measurement of mitochondrial membrane potential

The JC‐1 fluorescent probe was purchased from Beyotime Biotech (Shanghai, China). Chondrocytes were treated with or without H_2_O_2_ and various concentrations of Iso for 24 hours. The JC‐1 probe was administered according to the manufacturer's instructions. The fluorescence signals were measured using confocal fluorescence imaging microscopy. Mitochondrial depolarization occurring during apoptosis could be indicated as an increase in the green/red fluorescence ratio.^28^

### 2.12 Cell apoptosis detection by TUNEL staining

The TUNEL apoptosis detection kit was purchased from Beyotime Biotech (Shanghai, China). Primary chondrocytes were seeded in confocal dishes for 12 hours. The cells were treated with or without H_2_O_2_ and various concentrations of Iso for 24 hours. After fixation in 4% paraformaldehyde for 30 minutes, the cells were treated with 0.1% Triton X‐100 for 5 minutes. Then, the cells were sealed for 1 hour in a TUNEL reaction mixture at 37°C. The cells were incubated with DAPI for 5 minutes and observed with confocal fluorescence microscopy. The rate of TUNEL‐positive cells in each field was calculated.

### 2.13 Mouse model of knee OA induced by ACLT

Thirty 2‐month‐old male C57BL/6J mice were purchased from Shanghai SLAC Laboratory Animal Company (Shanghai, China) and then fed commercial food and water in specific pathogen‐free (SPF) conditions approved by the Animal Ethical Committee of Shanghai Ninth People's Hospital. ACLT was performed to induce abnormal mechanical loading‐associated OA of the right knee according to previous studies.^29^, ^30^ The anterior drawer test was performed to confirm complete transection. Following surgery, animal care was performed and each mouse was administered penicillin once every day during the first 3 days to prevent infection. A sham operation was conducted by opening the joint capsule and then suturing the incision. Thirty mice were randomly divided into five groups with six mice per group. Group 1 mice underwent a sham operation. For groups 2‐5, mice were subjected to ACLT of the right knee and mice in group 2 were injected intraperitoneally with PBS. Groups 3‐5 were injected intraperitoneally with Iso at concentrations of 10, 20 and 40 mg/kg bw every other day for 4 weeks.

### 2.14 Micro‐CT scanning

At the end of 4 weeks after injection, the knee joints of the mice were harvested and fixed in 4% paraformaldehyde. Specimens were scanned using micro‐CT (μCT 80; Scanco, Zurich, Switzerland) as described previously.^31^ The micro‐CT parameters were as follows: voltage, 70 kV; electric current, 114 μA; and resolution of 10 μm per pixel. Three‐dimensional structural parameters that were analysed included bone volume (BV), bone volume/total tissue volume (BV/TV), trabecular number (Tb.N), trabecular thickness (Tb.Th) and trabecular separation (Tb.Sp).

### 2.15 Histological observations

Samples were decalcified in 10% EDTA for 3 weeks and then embedded in paraffin. For microstructure observation, sagittal sections of the knee joint medial compartment were cut with a thickness of 4 μm and stained with haematoxylin and eosin (H&E), safranin O‐fast green (S&F) and TRAP. Osteoarthritis Research Society International (OARSI) scores were calculated as previously described.^32^ Moreover, TUNEL and MMP‐3 (Santa, USA; dilution 1:100) immunohistochemical staining was accomplished. Total and positively stained chondrocytes in the entire articular cartilage were calculated.

### 2.16 Statistical analysis

Each group contained three samples in vitro. All data were expressed as means ± standard deviations. One‐way analysis of variance (ANOVA) was used for multifactorial comparisons in this study. The assumption of normality was verified and the homogeneity of variance was tested. Differences between each group were assessed using post hoc multiple comparisons. Specifically, if there was no heterogeneity observed, the Bonferroni test was used to assess the differences between groups. However, if heterogeneity existed, the Welch test was used to test the equality of means and Dunnett's T3 test used to assess the differences. *P* < 0.05 were considered statistically significant. The following symbols were used to indicate statistical significance: * and ^#^ indicate *P* < 0.05, ** and ^##^ indicate *P* < 0.01. All data analysis was conducted using SPSS 22.0 analysis software (SPSS Inc, Chicago, IL).

## 3 RESULTS

### 3.1 Iso inhibits RANKL‐induced ROS production and osteoclastogenesis in vitro

**Figure 1:**
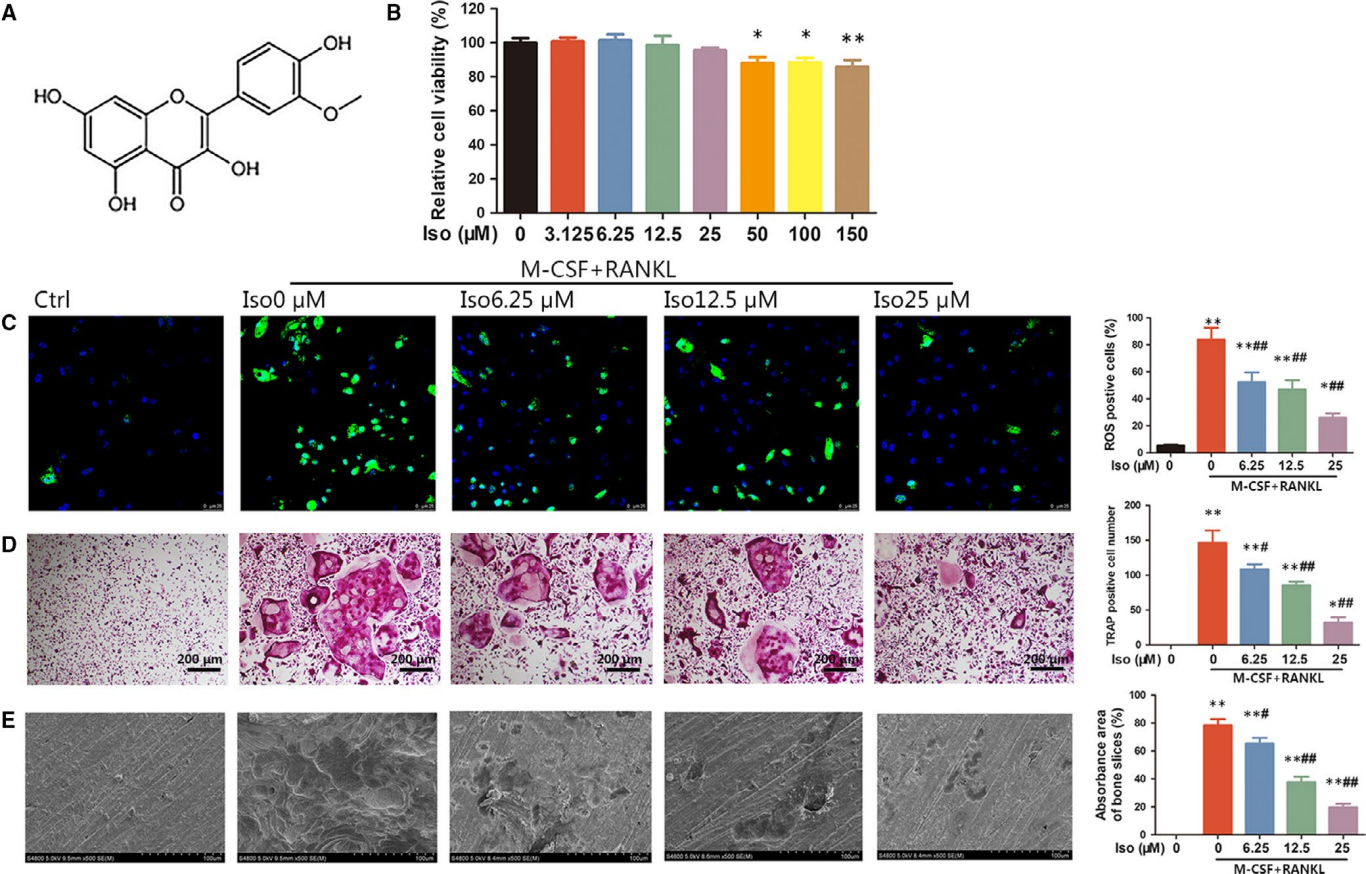
Iso inhibits receptor activator of nuclear factor‐kappa B ligand (RANKL)‐induced reactive oxygen species (ROS) production and osteoclastogenesis in vitro. (A) The chemical structure of Iso. (B) Bone marrow macrophages (BMMs) were treated with various concentrations of Iso for 24 h and analysed using a CCK‐8 assay. (C) Intracellular ROS levels of BMMs detected by DCFH‐DA probe. (D) Tartrate‐resistant acid phosphatase staining of osteoclasts treated with different concentrations of Iso. (E) Representative SEM images of bone resorption pits. *Compared with the control group and ^#^compared with the RANKL and M‐CSF without Iso group

The chemical structure of Iso is shown in Figure 1. To evaluate the influence of Iso on osteoclast formation in vitro, the cytotoxicity of Iso was first analysed using a CCK‐8 assay. The results showed that low concentrations of Iso (3.125, 6.25, 12.5 and 25 μmol/L) had minimal inhibitory effects, whereas 50, 100 and 150 μmol/L concentrations of Iso significantly inhibited cell viability at 24 hours (Figure 1). In RANKL‐treated BMMs, intracellular ROS levels were markedly increased compared with those of the control group. However, ROS generation was considerably inhibited with Iso treatment (Figure 1). Furthermore, BMMs were treated with M‐CSF (30 ng/mL), RANKL (50 ng/mL) and varying concentrations of Iso (0, 6.25, 12.5 or 25 μmol/L) until mature osteoclasts formed. As shown in Figure 1, numerous TRAP‐positive multinucleate osteoclasts formed in the RANKL‐treated group, whereas Iso suppressed osteoclast formation in a dose‐dependent manner.

To investigate whether Iso could attenuate osteoclast function in vitro, bovine bone slices were placed in a 96‐well plate with BMMs seeded on them. The BMMs were stimulated with M‐CSF and RANKL and varying concentrations of Iso. As shown in Figure 1, numerous resorption pits were observed in RANKL‐stimulated cells; however, Iso distinctly suppressed the formation of resorption pits. Quantitative analysis showed that approximately 80% of the area on the bone slice surface consisted of bone resorption pits in the RANKL‐stimulated group, whereas the resorption pit area was reduced to approximately 62, 38 and 18% in the 6.25, 12.5 and 25 μmol/L Iso‐treated groups (Figure 1) respectively. These results showed that osteoclast function was inhibited by Iso in vitro.

### 3.2 Iso inhibits the expression of osteoclastogenesis‐related genes and proteins in BMMs

**Figure 2:**
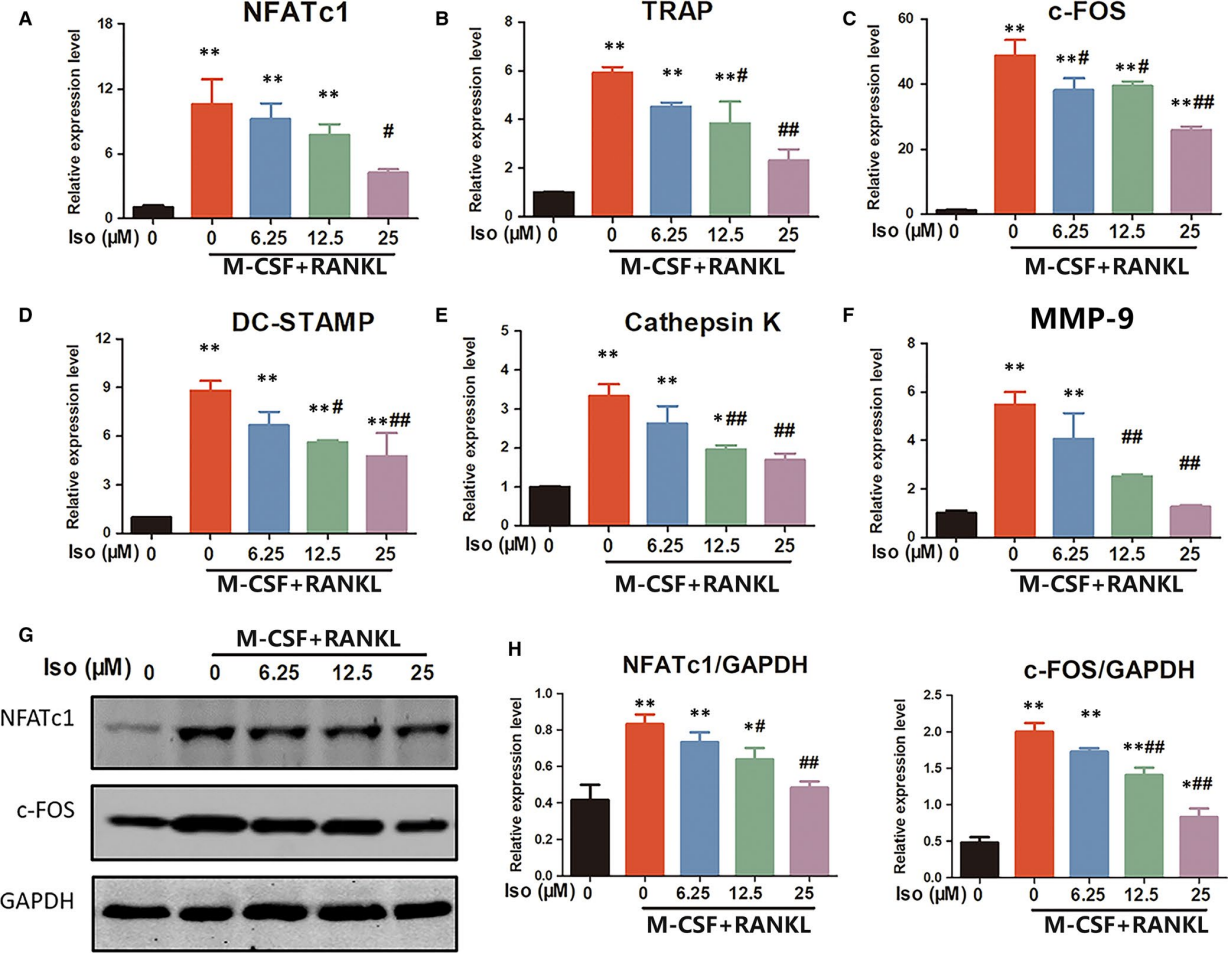
Iso suppresses receptor activator of nuclear factor‐kappa B ligand (RANKL)‐induced expression of osteoclastogenesis‐related genes and proteins. The expression of osteoclast‐specific mRNA, including (A) NFATc1, (B) tartrate‐resistant acid phosphatase, (C) c‐FOS, (D) DC‐STAMP, (E) cathepsin K, and (F) MMP‐9, was analysed using qRT‐PCR. (G) Western blotting was performed to analyse the expression of osteoclastogenesis‐related transcription factors including NFATc1 and c‐FOS. (H) Quantification of NFATc1 and c‐FOS expression levels. *Compared with the control group, and ^#^compared with the RANKL and M‐CSF without Iso group

Osteoclast differentiation is regulated by specifically related signalling pathways, which leads to the expression of particular genes.^33^ The expression of osteoclastogenesis‐related genes, including the nuclear factor of activated T‐cells 1 (NFATc1), TRAP, c‐FOS, DC‐STAMP, cathepsin K and MMP‐9, was analysed using quantitative real‐time polymerase chain reaction (qRT‐PCR). As shown in Figure 2, the expression of the mRNAs encoding osteoclast formation was markedly increased by RANKL. However, Iso significantly suppressed RANKL‐induced osteoclastogenesis‐related mRNA expression of these genes in a dose‐dependent manner. Western blotting results were consistent with the qRT‐PCR results, showing that RANKL stimulation dramatically up‐regulated the protein expression of NFATc1 and c‐FOS in BMMs. However, Iso clearly attenuated RANKL‐stimulated osteoclast‐related protein expression (Figure 2). These results confirmed the function of Iso in suppressing the expression of osteoclast‐specific genes and proteins in vitro.

### 3.3 Iso suppresses RANKL‐induced activation of MAPK/NF‐κB/AKT signalling pathways

**Figure 3:**
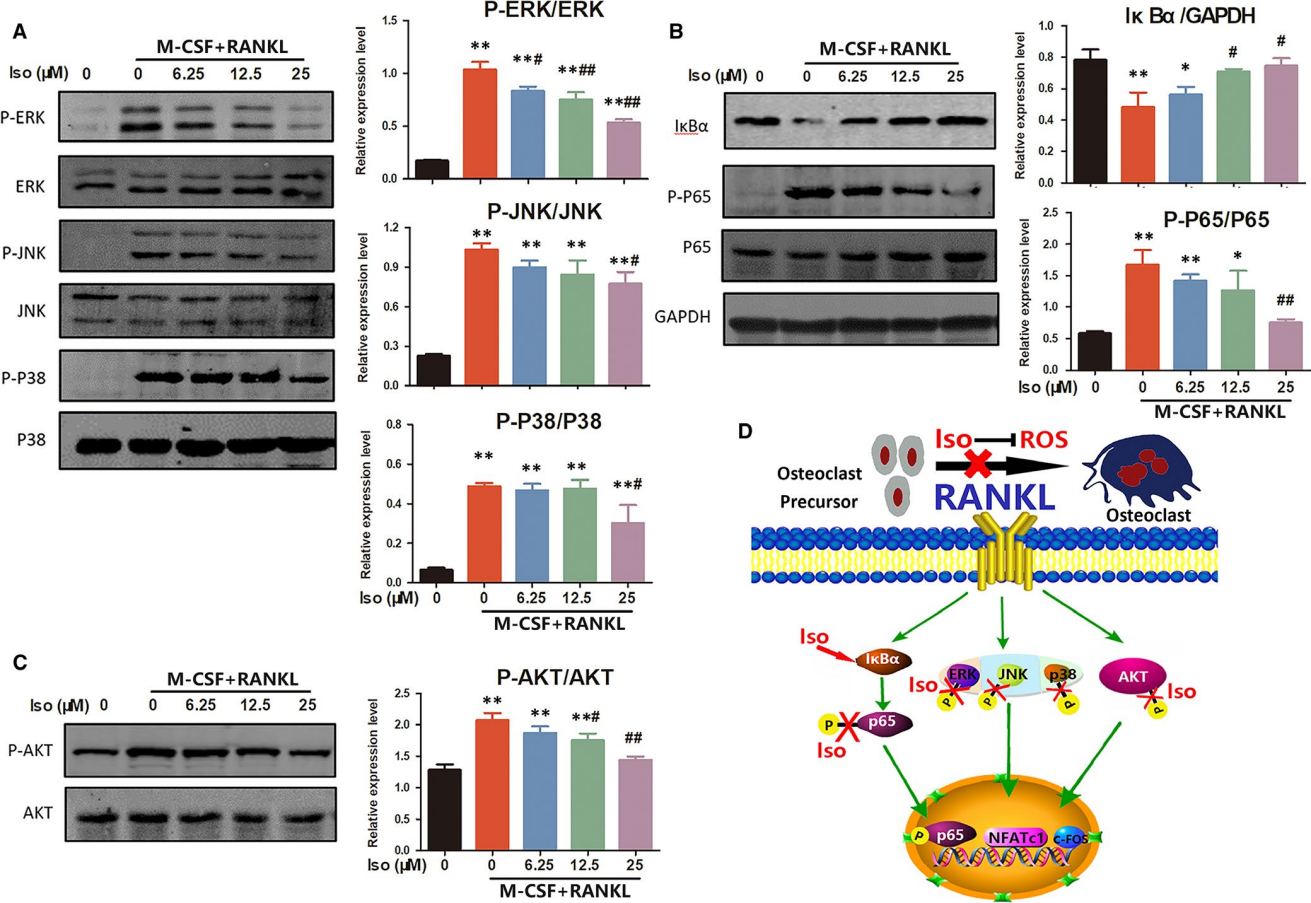
Iso treatment prevents osteoclastogenesis by regulating mitogen‐activated protein kinase (MAPK), NF‐κB and AKT signalling pathways. (A) The effects of Iso on MAPK signalling pathway proteins, including p‐ERK/ERK, p‐JNK/JNK and p‐p38/p38. (B) Western blotting showed that Iso affected the NF‐κB pathway‐related proteins IκBα, p65 and p‐p65. (C) The effects of Iso on AKT and the phosphorylation of AKT. *Compared with the control group and ^#^compared with the receptor activator of nuclear factor‐kappa B ligand and M‐CSF without Iso group. (D) A schematic illustration showing signalling pathways involved in osteoclast differentiation regulated by Iso

Binding of RANKL to the receptor RANK could activate the MAPK pathway.^34^ Three major MAPK signalling cascades, ERK, JNK and p38, have been shown to be crucial for activating osteoclast formation and function. As shown in Figure 3, RANKL treatment clearly promoted phosphorylation of ERK, JNK and p38. However, phosphorylation of ERK, JNK and p38 considerably decreased after Iso treatment. RANKL‐induced NF‐κB activation is essential for initiating osteoclast differentiation.^35^ As indicated in Figure 3, western blotting analysis showed that RANKL treatment drastically caused degradation of IκBα while increasing phosphorylation of p65. However, Iso up‐regulated IκBα expression and reduced phosphorylation of p65 in a dose‐dependent manner. In addition to the MAPK/NF‐κB pathway, the AKT‐NFATc1 signalling axis has also been reported to be significant in osteoclast formation.^27^ The results showed that RANKL treatment significantly increased phosphorylation of AKT in BMMs; however, the activation effects were substantially suppressed by Iso (Figure 3). All of these results indicated that Iso could attenuate osteoclast formation by inhibiting MAPK/NF‐κB/AKT pathways.

A schematic illustration shows the signalling pathways involved in osteoclast differentiation regulated by Iso in Figure 3. Iso inhibited RANKL‐induced ROS generation. Moreover, Iso targeted the NF‐κB pathway‐related protein IκBα and inhibited downstream p65 phosphorylation, which further affected nuclear translocation of phosphorylated p65. Iso treatment also decreased phosphorylation of ERK, JNK and p38, indicating that Iso could lead to inhibition of the MAPK pathway. Furthermore, Iso suppressed phosphorylation of AKT. Overall, this activity resulted in decreased expression levels of transcription factors such as NFATc1 and c‐FOS, which inhibited osteoclast precursor cells from differentiating into osteoclasts.

### 3.4 The direct effects of Iso on chondrocytes

To investigate the direct effects of Iso on chondrocytes, a CCK‐8 assay was first used to evaluate the cytotoxic effects of Iso. As shown in Figure S1A, the concentrations of Iso used to inhibit osteoclast formation (6.25, 12.5, 25 μmol/L) had minimal inhibitory effects on chondrocytes. Cell cycle analysis showed that Iso treatment did not noticeably affect the cell cycle of chondrocytes (Figure S1B). Furthermore, Iso treatment had a minimal effect on the collagen II expression of chondrocytes (Figure S1C). All of these results indicated that Iso treatment did not directly affect chondrocytes. Moreover, the viability of chondrocytes after various concentrations of H_2_O_2_ treatment was measured using CCK‐8 at 24 hours. The results demonstrated that chondrocyte viability was significantly (*P* < 0.01) suppressed by H_2_O_2_ at a concentration of 200 μmol/L (Figure S1D). Therefore, 200 μmol/L H_2_O_2_ was used for subsequent experiments in chondrocytes.

### 3.5 Iso protects chondrocytes from ROS‐induced apoptosis

**Figure 4:**
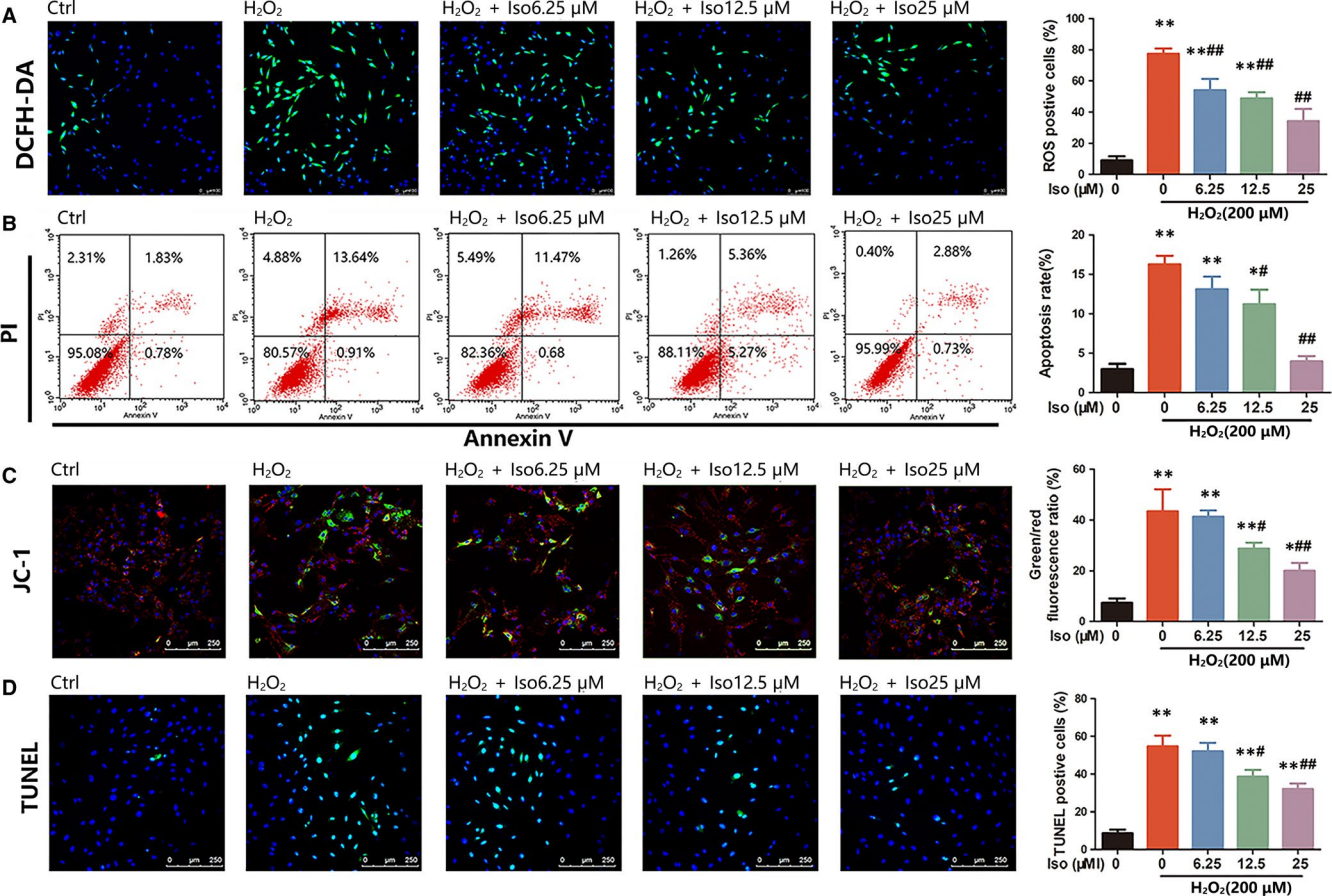
Iso inhibits reactive oxygen species (ROS)‐induced chondrocyte apoptosis. (A) The production of intracellular ROS detected by DCFH‐DA probe. (B) Primary chondrocyte apoptosis assessed using flow cytometry analysis. (C) Measurement of mitochondrial membrane potential by JC‐1. (D) TUNEL staining to assess cell apoptosis. *Compared with the control group and ^#^compared with the H_2_O_2_ without Iso group

The production of intracellular ROS was detected by DCFH‐DA probe. We found that the number of ROS‐positive cells was clearly increased in chondrocytes treated with H_2_O_2_ for 24 hours. However, Iso could alleviate the production of ROS stimulated by H_2_O_2_ in chondrocytes (Figure 4). In addition, in the H_2_O_2_‐treated group, the apoptosis rate was 16.26 ± 1.88%, which was reduced to 13.07 ± 2.82%, 11.17 ± 3.26% and 3.94 ± 1.18% in the Iso 6.25, 12.5 and 25 μmol/L treated groups respectively, showing that Iso effectively decreased the apoptosis rate caused by H_2_O_2_ (Figure 4). JC‐1 staining showed a significant increase in the green/red ratio in H_2_O_2_‐treated cells compared with the control group, whereas the ratio was reduced by Iso in a dose‐dependent manner (Figure 4). These results were also confirmed by TUNEL staining, showing that in the H_2_O_2_‐treated group, the percentage of TUNEL‐positive cells was 54.67 ± 9.8%, whereas it was reduced to 51.66 ± 6.1%, 38.7 ± 5.30%, 32.3 ± 6.7% in the Iso‐treated (6.25, 12.5 and 25 μmol/L) groups (Figure 4). All results indicated that Iso reduced ROS‐induced chondrocyte apoptosis.

### 3.6 Iso inhibits chondrocyte apoptosis by modulating apoptosis‐related genes and proteins

**Figure 5:**
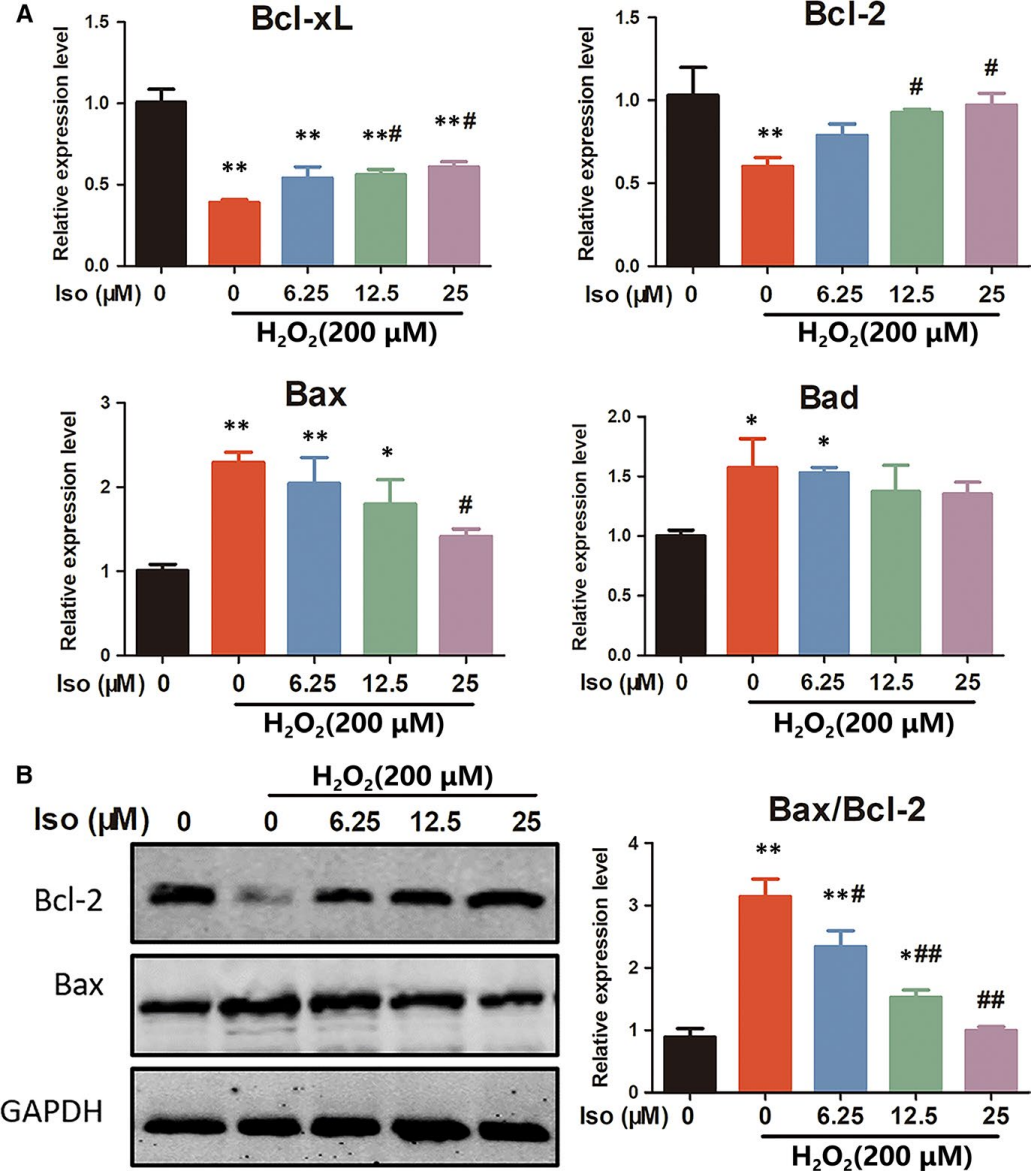
Iso inhibits chondrocyte apoptosis by modulating apoptosis‐related genes and proteins. (A) The expression of apoptosis‐related genes, including Bcl‐xL/Bcl‐2 (anti‐apoptotic) and Bax/Bad (pro‐apoptotic), was evaluated using qRT‐PCR. (B) Western blotting showed the effects of Iso on apoptosis‐related proteins Bcl‐2 and Bax. *Compared with the control group and # compared with the H_2_O_2_ without Iso group

qRT‐PCR results showed that the mRNA expression of Bcl‐xL and Bcl‐2 (anti‐apoptotic proteins) was down‐regulated, whereas Bax and Bad (pro‐apoptotic proteins) were up‐regulated after H_2_O_2_ stimulation. However, Iso treatment not only promoted the expression of Bcl‐xL and Bcl‐2 but also inhibited the expression of Bax and Bad (Figure 5). These results were consistent with the western blotting results; the Bax/Bcl‐2 ratio increased after H_2_O_2_ stimulation, whereas Iso dose‐dependently decreased the ratio (Figure 5). These results signified that Iso reduced ROS‐induced chondrocyte apoptosis by modulating apoptosis‐related proteins.

### 3.7 Iso suppresses subchondral bone loss induced by ACLT in vivo

**Figure 6:**
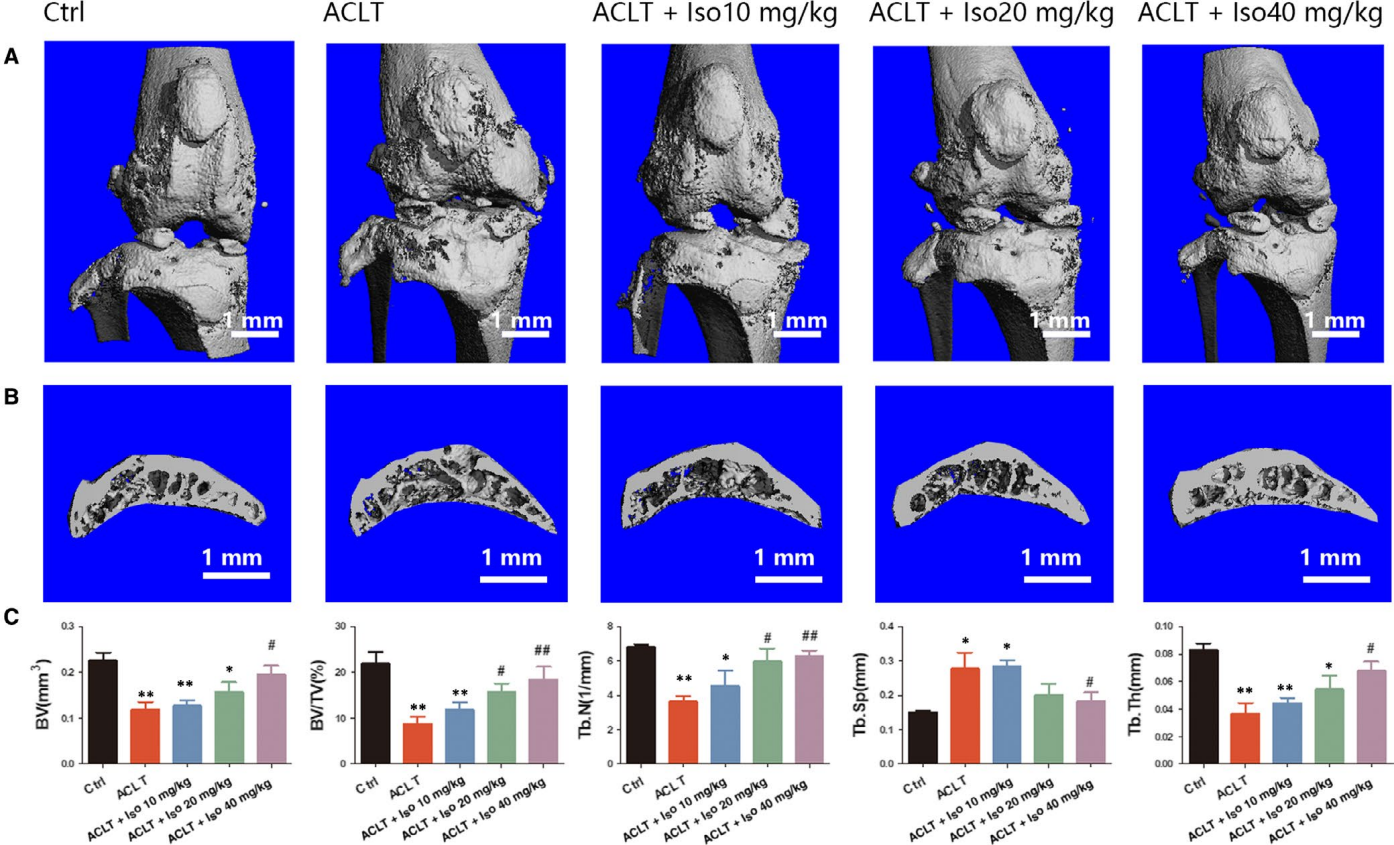
Micro‐CT analysis of Iso‐treated OA induced by anterior cruciate ligament transection (ACLT). (A) Three‐dimensional μCT images of frontal views of the knee joints at 4 wk after the sham operation or the ACLT operation. (B) Sagittal views of the medial compartment of subchondral bone. (C) Quantitative analysis of BV, BV/TV, Tb.N, Tb.Sp and Tb.Th. *Compared with the control group and ^#^compared with the ACLT group

To investigate the effects of Iso on osteoclasts during the development of OA, we administered Iso intraperitoneally in ACLT‐induced mice. As shown in Figure 6, ACLT injury induced significant bone resorption in the subchondral bone as shown by the increased area of bone lesions in the μCT results. Nonetheless, the destruction was reduced by Iso treatment dose‐dependently, showing increased integrity of the knee bone. Moreover, sagittal views of the medial compartment of subchondral bone showed that Iso decreased ACLT‐induced bone resorption, indicating that osteolysis was diminished after Iso treatment (Figure 6). Statistically, the tibial plateau BV was significantly less than that of the control group. Nonetheless, BV was clearly up‐regulated in 10, 20 and 40 mg/kg Iso‐treated mice. BV and other parameters, including BV/TV, Tb.N, Tb.Th and Tb.Sp demonstrated that Iso suppressed bone resorption in vivo (Figure 6).

### 3.8 Iso inhibits osteoclasts and protects chondrocytes in vivo

**Figure 7:**
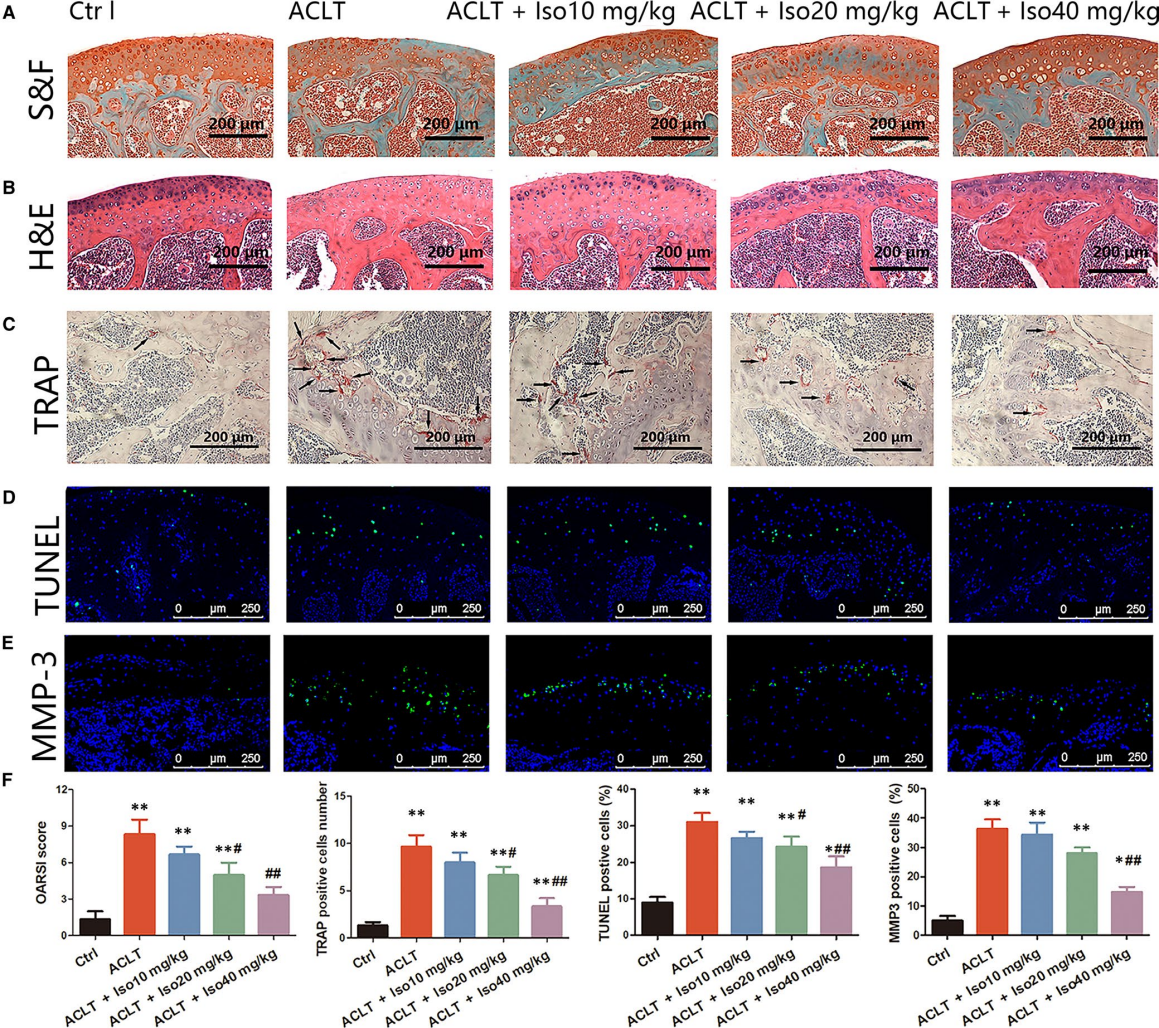
Staining analysis of Iso treatment in anterior cruciate ligament transection (ACLT)‐induced osteoarthritis. Knee joint medial compartment cartilage and subchondral bone were stained by (A) S&F and (B) H&E. (C) Osteoclasts in subchondral bone were stained by tartrate‐resistant acid phosphatase. (D‐E) TUNEL and MMP‐3 staining of chondrocytes in articular cartilage. (F) Quantitative analysis of the Osteoarthritis Research Society International score and positively stained cells in articular cartilage. *Compared with the control group and ^#^compared with the ACLT group

S&F and H&E staining demonstrated the loss of proteoglycan and decreased thickness of articular cartilage induced by ACLT surgery. However, in Iso‐treated mice, degeneration of cartilage was inhibited significantly (Figure 7). Moreover, an increased number of mature osteoclasts (TRAP‐positive cells) were observed in the ACLT group, whereas Iso treatment showed a decreased number of mature osteoclasts (Figure 7). Additionally, Iso inhibited chondrocyte apoptosis (TUNEL‐positive chondrocytes) and MMP‐3 expression in articular cartilage (Figure 7). Quantitative analysis showed that the progression of OA was delayed by Iso and the numbers of osteoclasts and TUNEL‐ and MMP‐3‐positive cells were distinctly reduced after Iso treatment (Figure 7). These data showed that Iso remedied cartilage degeneration and attenuated OA progression.

## 4 DISCUSSION

In this study, an ACLT‐induced OA model was used to evaluate the effects of Iso treatment. ACLT and destabilization of the medial meniscus (DMM) are two surgically induced OA models that have been mostly used. However, thinning of the subchondral plate in the early stage of OA has only been shown in the ACLT‐induced OA model; hence, we used an ACLT‐induced OA model to better observe changes in subchondral bone.^36^, ^37^ We demonstrated that Iso preserved subchondral bone microarchitecture by decreasing osteoclasts and inhibiting bone resorption. Additionally, Iso suppressed chondrocyte apoptosis and protected articular cartilage from degeneration. All of these findings signified that Iso was an effective treatment for OA.

In vitro, we demonstrated that Iso inhibited osteoclast formation and function. Iso suppressed RANKL‐induced ROS generation, which is an important mediator for osteoclastogenesis. Furthermore, we proved that Iso suppressed osteoclastogenesis‐related genes and proteins and inhibited the MAPK/NF‐κB/AKT signalling pathway activated by RANKL. Iso was also found to protect chondrocytes from ROS‐induced apoptosis.

ROS have been speculated to activate several molecules involved in RANKL‐induced osteoclastogenesis. Lee et al reported that ROS produced by the NADPH oxidases Nox1 and Nox2 participated in osteoclast differentiation.^38^ Moreover, RANKL‐induced ROS generation mediates NFATc1 activation and further osteoclastogenesis by stimulating phospholipase C gamma 1 (PLCg1).^39^ Our results demonstrated that RANKL stimulation markedly increased levels of intracellular ROS, which were inhibited by Iso treatment in a dose‐dependent manner.

H_2_O_2_ formed from superoxide anions through superoxide dismutase can diffuse through the mitochondrial membranes into the cytosol and further induce oxidative stress.^40^ Thus, H_2_O_2_ is frequently used as an active stimulus to mimic the oxidative stress microenvironment in vitro.^41^ H_2_O_2_ clearly promoted the production of ROS, which could further induce the leakage of cytochrome c and hence cell apoptosis.^42^ We found that H_2_O_2_ significantly induced chondrocyte apoptosis at a concentration of 200 μmol/L as evaluated using annexin V/ PI, JC‐1 and TUNEL staining. These data were consistent with the PCR and western blotting results, which showed that H_2_O_2_ distinctly up‐regulated the pro‐apoptotic proteins Bax and Bad while down‐regulating the anti‐apoptotic proteins Bcl‐xL and Bcl‐2. However, Iso could effectively decrease intracellular ROS levels further to prevent chondrocytes from undergoing apoptosis.

Several signalling pathways have been shown to be related to osteoclast formation. The classical NF‐κB pathway involves phosphorylation and degeneration of IκBα, which further promotes phosphorylation of the NF‐κB protein, including the p65 subunit. Phosphorylated p65 translocates into the nucleus and binds to specific DNA sites to activate the expression of osteoclastogenesis‐related genes.^43^ Our results demonstrated that phosphorylation of p65 was markedly activated by RANKL stimulation in BMMs, whereas Iso suppressed NF‐κB activation as indicated by the increased expression level of IκBα and decreased phosphorylation of p65. NFATc1 has been shown to be a key transcription factor that plays an important role in osteoclastogenesis.^44^ Moreover, NFATc1 can activate varying osteoclastogenic genes associated with osteoclast differentiation and function, including c‐FOS, TRAP, MMP‐9, cathepsin K and DC‐STAMP.^45^ The AKT‐NFATc1 signalling axis is important in osteoclast formation and it has been reported that inhibition of AKT activation could prevent RANKL‐induced osteoclastogenesis.^46^ In the present study, we found that Iso dramatically suppressed the phosphorylation of AKT activated by RANKL in BMMs. MAPK is in the serine‐threonine protein kinase family that is involved in cell viability, differentiation, apoptosis, inflammation and innate immunity.^47^ The most studied proteins of the MAPK pathway are ERK, JNK and p38 kinases, which can respond to extracellular stimuli including growth factors, heat shock proteins and pro‐inflammatory cytokines.^48^ Specific inhibitors of JNK, p38 and ERK can inhibit RANKL‐stimulated osteoclast differentiation, indicating that MAPK is important for osteoclast activation and bone resorption.^49^, ^50^ Our data showed that Iso clearly suppressed RANKL‐induced phosphorylation of ERK in a dose‐dependent manner. Interestingly, Iso clearly inhibited the phosphorylation of JNK and p38 compared with the RANKL‐stimulation group only at the concentration of 25 μmol/L, indicating that the pharmacological effects of Iso are concentration‐dependent. These data suggested that Iso reduced RANKL‐induced osteoclastogenesis by regulating the MAPK/NF‐κB/AKT signalling pathway.

In this study, we revealed the vital role of ROS in the progression of OA and for the first time, proved that Iso could not only suppress osteoclastogenesis but also protect chondrocytes by modulating ROS. This finding could provide a novel theoretical basis for OA treatment. However, this study also has potential limitations. First, we just signified the effects of Iso on the MAPK/NF‐κB/AKT signalling pathway; whether Iso can affect other mediators and signalling pathways involved in osteoclastogenesis is unknown. Moreover, the cells used in this study were obtained from mice instead of humans; hence, it is necessary to further investigate the effect of Iso on human cells for translational use.

In conclusion, our investigation showed the beneficial effects of Iso in treating OA. Iso inhibited RANKL‐induced osteoclastogenesis and protected chondrocytes by modulating ROS. All the data indicated that Iso could be a potential agent for the treatment of OA.

## CONFLICT OF INTEREST

The authors confirm that there are no conflicts of interest.

## AUTHORS' CONTRIBUTIONS

F. Zhou and J.T. Mei designed the research. F. Zhou, J.T. Mei, K. Yuan and X.G. Han conducted the cell experiments and performed the animal surgeries. F. Zhou prepared the manuscript. T.T. Tang and H. Qiao revised the manuscript content.
