# Supplementary material for: Isorhamnetin attenuates osteoarthritis by inhibiting osteoclastogenesis and protecting chondrocytes through modulating reactive oxygen species homeostasis
Source: J Cell Mol Med. 2019 Apr 14;23(6):4395–407. doi: 10.1111/jcmm.14333 (PMC6533508; doi:10.1111/jcmm.14333)
Supplement: Supplementary file 1 [file JCMM-23-4395-s001.docx]

**Table S1** Primer sequences used in the article**.**

| GAPDH-F | 5’-ACCCAGAAGACTGTGGATGG-3’ |
| --- | --- |
| GAPDH-R | 5’-CACATTGGGGGTAGGAACAC-3’ |
| NFATc1-F | 5’-CCGTTGCTTCCAGAAAATAACA-3’ |
| NFATc1-R | 5’-TGTGGGATGTGAACTCGGAA-3’ |
| TRAP-F | 5’-CTGGAGTGCACGATGCCAGCGACA-3’ |
| TRAP-R | 5’-TCCGTGCTCGGCGATGGACCAGA-3’ |
| c-Fos-F | 5’-GTTCGTGAAACACACCAGGC-3’ |
| c-Fos-R | 5’-GGCCTTGACTCACATGCTCT-3’ |
| DC-STAMP-F | 5’-AAAACCCTTGGGCTGTTCTT-3’ |
| DC-STAMP-R | 5’-AATCATGGACGACTCCTTGG-3’ |
| cathepsin K-F | 5’-TCCGCAATCCTTACCGAATA-3’ |
| cathepsin K-R | 5’-AACTTGAACACCCACATCCTG-3’ |
| MMP9-F | 5’- GCGTCATTCGCGTGGATAAG-3’ |
| MMP9-R | 5’- TGGAAACTCACACGCCAGAA-3’ |
| Bcl-xL-F | 5’-TTCGGGATGGAGTAAACTGGG-3’ |
| Bcl-xL-R | 5’-AGTCATGCCCGTCCACAAAA-3’ |
| Bcl-2-F | 5’-ATGCCTTTGTGGAACTATATGGC-3’ |
| Bcl-2-R | 5’-GGTATGCACCCAGAGTGATGC-3’ |
| Bax-F | 5’-TGAAGACAGGGGCCTTTTTG-3’ |
| Bax-R | 5’-AATTCGCCGGAGACACTCG-3’ |
| Bad-F | 5’-AAGTCCGATCCCGGAATCC-3’ |
| Bad-R | 5’-GCTCACTCGGCTCAAACTCT-3’ |


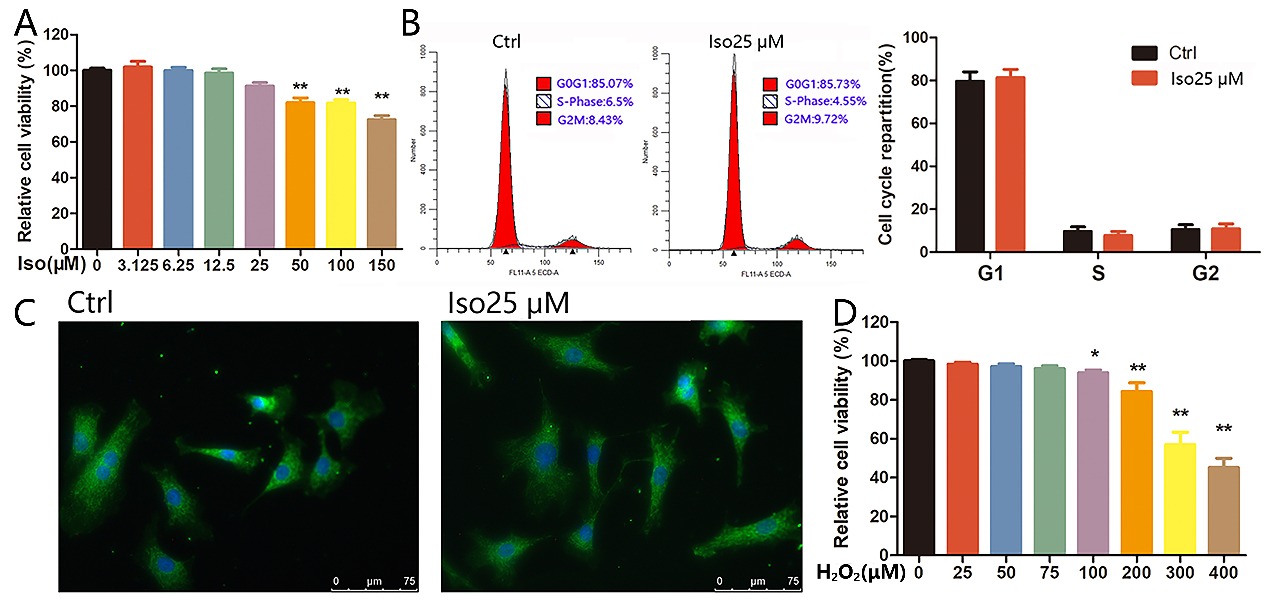


**Figure S1** The direct effects of Iso on chondrocytes. (A) Primary chondrocytes were treated with various concentrations of Iso for 24 h, and cell viability was analyzed using CCK-8 assay. (B) The cell cycle of chondrocytes after Iso treatment was analyzed by PI staining. (C) The expression of collagen II in chondrocytes was analyzed by immunofluorescence staining. (D) Chondrocytes were treated with various concentrations of H_2_O_2_ for 24 h, cell viability was analyzed using CCK-8 assay. * compared with the control group.
